# Effect of long-term rotation on astigmatism following EVO-toric intraocular collamer lens implantation

**DOI:** 10.3389/fmed.2023.1194006

**Published:** 2023-07-14

**Authors:** Xun Chen, Huamao Miao, Mingrui Cheng, I-Chun Lin, Boliang Li, Yinjie Jiang, Yadi Lei, Xiaoying Wang, Xingtao Zhou

**Affiliations:** ^1^Fudan University Eye Ear Nose and Throat Hospital, Shanghai, China; ^2^National Health Commission Key Lab of Myopia (Fudan University), Shanghai, China; ^3^Shanghai Research Center of Ophthalmology and Optometry, Shanghai, China; ^4^Shanghai Engineering Research Center of Laser and Autostereoscopic 3D for Vision Care (20DZ2255000), Shanghai, China

**Keywords:** EVO-toric intraocular collamer lens implantation, astigmatism, moderate and high myopia, OPD-scan, rotation

## Abstract

**Objective:**

To evaluate the effect of long-term rotation on astigmatism following Evolution-toric intraocular collamer lens (EVO-TICL) implantation.

**Methods:**

Forty eyes of 22 patients were enrolled in this prospective study. Visual acuity, refractive parameters, and axial position of the EVO-TICL by OPD-Scan III aberrometer were measured preoperatively, 1 month and 3 years postoperatively.

**Results:**

Last visit, the safety index was 1.32 ± 0.15 and the efficacy index was 1.01 ± 0.23. The best-fitting curve of the attempted versus achieved correction was y = 0.9751x + 0.001. The mean spherical equivalent (SE) decreased from −8.94 ± 2.72D preoperatively to 0.06 ± 0.24D and − 0.36 ± 0.46D 1 month and 3 years postoperatively, respectively. The mean target and surgical induced astigmatism were 1.55 ± 0.61D and 1.67 ± 0.94D 3 years postoperatively. The average expected axis of the TICL was-1.15 ± 9.07 (−21–19°). One month and 3 years postoperatively, the average actual axis was −0.70 ± 9.86 (−20–20°) and − 0.35 ± 11.72 (−25–30°), respectively. The absolute rotation of the TICL was 3.70 ± 4.42 (0–22°) and 6.00 ± 6.70 (0–32°) 1 month and 3 years postoperatively, respectively (*p* < 0.001). The expected astigmatism was −0.10 ± 0.12D, and the mean actual astigmatism was −0.21 ± 0.30D and − 0.44 ± 0.45D 1 month and 3 years postoperatively, respectively. The mean absolute rotation without postoperative astigmatism was 3.73 ± 2.69 (0–9°) and 1.67 ± 1.66 (0–5°) for low (<2D) and high (≥2D) astigmatic TICL, respectively (*p* < 0.05).

**Conclusion:**

EVO-TICL implantation is safe and effective, with good predictability and stability. OPD-Scan is a fast device to detect the axial position of the TICL without mydriasis, and the axial position is relatively stable in the long term postoperatively. A slight rotation of low-astigmatic TICL may not cause postoperative astigmatism, whereas rotation of the high-astigmatic TICL may cause it.

## Introduction

Myopia is a global health problem (1). Several studies have shown that the prevalence of astigmatism in the population can reach >30% and that people with myopia are more likely to suffer from astigmatism (2, 3). Even 0.75 D uncorrected astigmatism can cause significantly decreased vision (4); therefore, precise correction of astigmatism has always been the focus of attention.

Evolution-toric intraocular collamer lens (EVO-TICL; STAAR Surgical, Monrovia, CA), a posterior chamber phakic intraocular lens, has been widely used in recent years because of its wide range of correction of myopia and astigmatism, independent of corneal conditions. The safety, effectiveness, stability, predictability, and significant improvement in postoperative visual quality have been reported previously (5, 6). Accurate operative alignment of the TICL axis and its stable postoperative position are crucial for achieving ideal refractive outcomes (7). Once EVO-TICL rotates, it may cause new astigmatism and visual impairment. To our knowledge, few studies have reported the short-term efficacy and safety of EVO-TICL implantation for myopic astigmatism correction (8, 9), and our study is the first to prospectively investigate the long-term efficacy, safety, and rotational stability, and the effect of rotation on astigmatism of EVO-TICL.

## Materials and methods

### Study population

This prospective study was approved by the Ethics Committee of Fudan University Eye, Ear, Nose, and Throat Hospital. Patients participated voluntarily and signed an informed consent form. This study included 40 eyes of 22 patients who underwent EVO-ICL implantation. The male-to-female ratio was 3:19, the average age was 24.82 ± 4.32 (18–33) years old, the preoperative equivalent spherical lens was −8.94 ± 2.72 (−1.38–15.75) D, and the preoperative average cylindrical lens was −1.60 ± 0.61 (−0.75–3.00) D (Table 1).

**Table 1:** Distribution of preoperative characteristics.

Parameter	Mean ± SD (range)
*N*, eyes	40
Age, years	24.82 ± 4.32 (18 ~ 33)
Gender (male:female)	3:19
logMAR CDVA	−0.02 ± 0.04 (−0.08 ~ 0.10)
**Refractive errors (D)**	
Spherical	−8.14 ± 2.77 (−0.50 ~ −15.25)
Cylindrical	−1.60 ± 0.61 (−0.75 ~ −3.00)
Spherical equivalent	−8.94 ± 2.72 (−1.38 ~ −15.75)
**Keratometric value (D)**	
Flat K	43.14 ± 1.18 (41.20 ~ 45.60)
Steep K	44.99 ± 1.04 (43.00 ~ 46.90)
WTW diameter (mm)	12.02 ± 0.34 (11.30 ~ 12.60)
IOP (mm Hg)	16.19 ± 2.08 (12.20 ~ 20.00)
CCT (mm)	522.43 ± 40.42 (452.00 ~ 612.00)
ACD (mm)	3.27 ± 0.24 (2.79 ~ 3.71)
Axial length (mm)	26.91 ± 1.37 (24.43 ~ 31.86)
ECD (cells/mm2)	2604.98 ± 191.18 (2146.00 ~ 2987.00)

*N*, number of eyes; UDVA, uncorrected distance visual acuity; CDVA, corrected distance visual acuity; D, diopters; K, keratometry; WTW, horizontal white-to-white diameter; IOP, intraocular pressure; CCT, central corneal thickness; ACD, anterior chamber depth; ECD, corneal endothelial cell density.

The inclusion criteria were as follows: (1) patients stopped wearing contact lenses for >1 week preoperatively, (2) the refraction was basically stable, with an annual growth of <0.50 D for at least 2 years, (3) the anterior chamber depth (ACD) was >2.8 mm, (4) the endothelial cell density was >2000 cells/mm^2^, (5) astigmatism ≥0.75 D.

The exclusion criteria were as follows: (1) eye inflammation, obvious refractive interstitial opacity, previous eye surgery, glaucoma, cataract, and other eye diseases, (2) systemic connective tissue and autoimmune diseases, such as systemic lupus erythematosus, rheumatoid arthritis, multiple sclerosis, and diabetes, (3) patients with mental or psychological abnormalities and unrealistic expectations.

Observation indicators and follow-up: Uncorrected distance visual acuity (UDVA), corrected distance visual acuity (CDVA, fully-automatic computer comprehensive refractometer, Nidek, Japan), refraction (fully-automatic computer comprehensive refractometer, Nidek, Japan), ocular axis (IOL Master, CarlZeiss, Germany), intraocular pressure (non contact tonometer, Canon, Japan), endothelial cell density (SP. 3000P, Topcon, Japan), vault (Pentacam AXL, Oculus, Germany), TICL axis (OPD-Scan III, Nidek, Japan), and 3-year follow-up. Each measurement was performed thrice by experienced technicians.

### TICL axis position measurement

**Figure 1:**
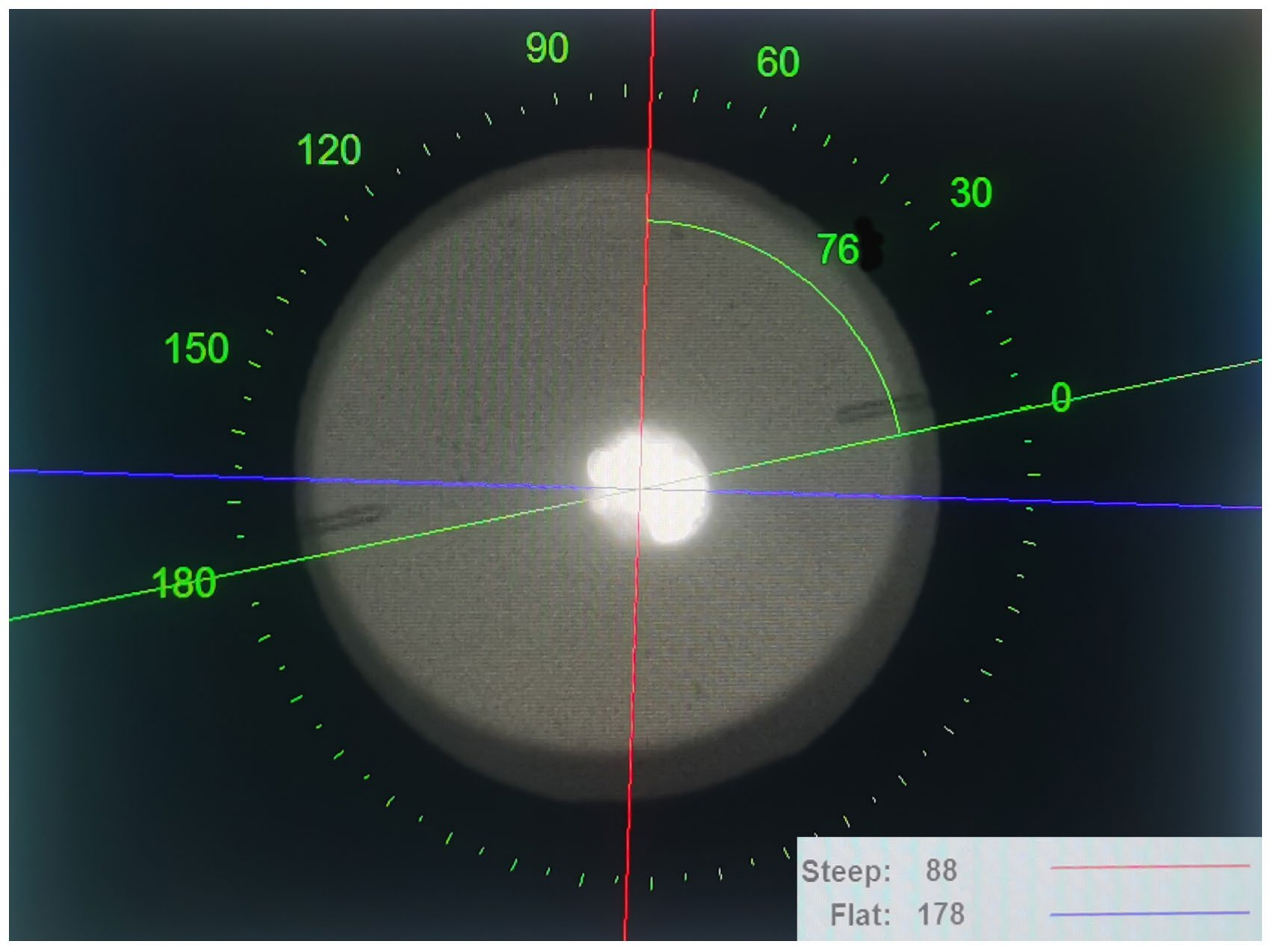
An image of the EVO-TICL, steep and flat axes measured by OPD-Scan III (Nidek Co., Ltd., Gamagori, Japan). The EVO-TICL axis can be calculated by the angle between the adjusted axis (the green line) and the calibration axis (the red line) using the image obtained from OPD-Scan III. For this case, the EVO-TICL axis = 88°–76° = 12°. EVO-TICL, Evolution-toric intraocular Collamer lens.

OPD-Scan III (Nidek Co., Ltd., Gamagori, Japan) was used to measure patients with TICL implantation preoperatively, 1 month postoperatively, and 3 years postoperatively, and to record the axial position of the TICL in the eye within serveral seconds without mydriasis. Using the image obtained by OPD-Scan III, the axis position was adjusted to be parallel to the TICL axis position, and the angle between the adjusted axis position and the calibration axis was calculated to determine the TICL axis position (Figure 1). Each measurement was performed thrice by experienced technicians, and the average of the three measurements was used in the analysis. Rotation was defined as the difference between the expected and actual axes at each follow-up.

### Surgical methods

All surgeries were performed with standard practice by the same surgeon (XW). The implanted intraocular lens was EVO-TICL, the optical area was 4.9–5.8 mm, the spherical lens power range was −0.5 to −18.0 D, and the cylindrical lens power range was +0.5 to +6 D. Four sizes were available: 12.1, 12.6, 13.2 and 13.7 mm.

Anti-inflammatory eyedrops were administered 3 days preoperatively. The mydriasis was started 30 min before ICL implantation, surface anesthesia was given to the operative eye, the cloth was disinfected routinely, the eyelid opener was used to open the eyelid, and a 3.0 mm corneal incision was made at the temporal corneoscleral margin. The special injector implanted the ICL in the crystal chamber into the eye. After the ICL unfolded naturally, a special positioning hook was used to adjust the haptics to the back of the iris. Postoperatively, the patients were administered the steroid drug brigitte (1% prednisolone acetate, Elgin, Ireland) qid * 3 days; antibacterial drugs (levofloxacin, Towering, Japan) qid * 1 week; non-steroidal anti-inflammatory drugs (Prauprofen, Ginseng, Japan) qid * 2 weeks; and artificial tear, qid * 1 month.

### Statistical analysis

SPSS 26.0 (SPSS Inc., IBM, United States) was used to analyze the data. The Kolmogorov–Smirnov test was used to test the normal distribution of data and repeated-measures analysis of variance was used to compare the data at various time points pre- and post-operatively. Also, a paired t-test was used to compare the parameters in the early and long postoperative period, and an independent sample t-test was used to compare low-and high-astigmatic TICL. Continuous variables were expressed as mean ± standard deviation. *p* < 0.05 was considered statistically significant.

## Results

### Study population

Forty eyes of 22 patients with preoperative manifest spherical equivalent (SE) and cylinder of −8.94 ± 2.72 and − 1.60 ± 0.61 D were included. Their mean age was 24.82 ± 4.32 (18–33) years. The preoperative data for the two groups are listed in Table 1.

### Efficacy

**Figure 2:**
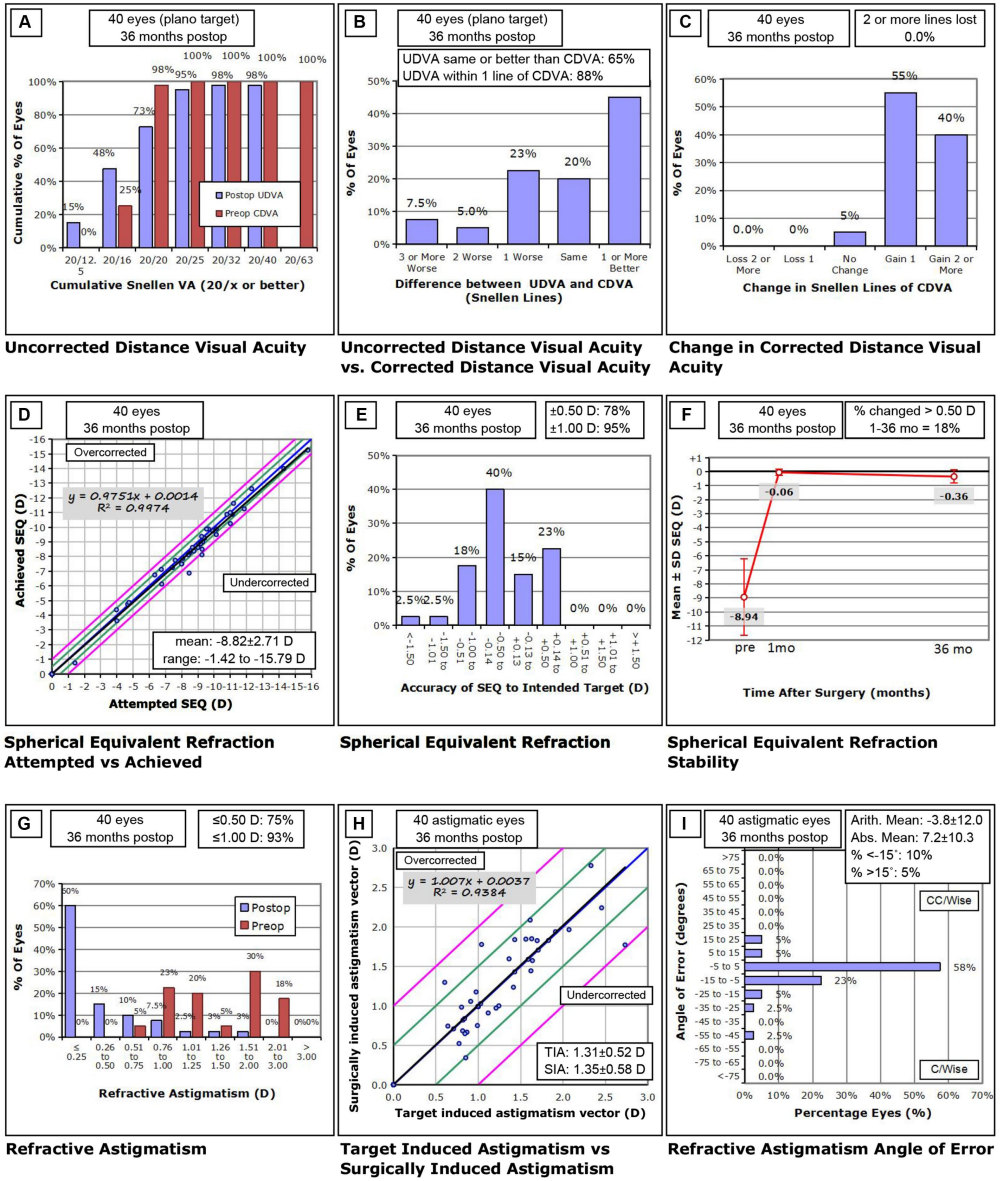
Standard graphs for reporting refractive surgery outcomes 36 months after Toric implantable collamer lens Implantation. **(A)** Uncorrected distance visual acuity (UDVA), **(B)** UDVA versus corrected distance visual acuity (CDVA), **(C)** change in CDVA, **(D)** spherical equivalent refraction (SEQ) attempted versus achieved, **(E)** spherical equivalent refraction accuracy, **(F)** spherical equivalent refraction stability, **(G)** refractive astigmatism, **(H)** target induced astigmatism (TIA) versus surgically induced astigmatism (SIA), **(I)** refractive astigmatism angle of error. D, diopters; preop, preoperative; postop, postoperative.

Three years postoperatively, UDVA was −0.01 ± 0.13 logMAR (range: −0.18–0.40 logMAR) and the efficacy index (postoperative CDVA/preoperative CDVA) was 1.01 ± 0.23. After 3 years, 97.50 and 65.00% of the eyes achieved UDVA of 20/40 and 20/20 or better, respectively (Figure 2A). Figure 2B shows the Snellen line differences between postoperative UDVA and preoperative CDVA; 65.00% of eyes achieved UDVA that was equal to or better than the preoperative CDVA.

### Safety

Three years postoperatively, CDVA was −0.13 ± 0.06 logMAR (range: −0.18–0.10 logMAR) and the safety index (postoperative CDVA/preoperative CDVA) was 1.32 ± 0.15. A significant increase was observed from the preoperative to the postoperative CDVA (*p* < 0.001). Figure 2C shows the changes in the CDVA Snellen line. No eye lost CDVA, 55.00% gained one line, 40.00% gained two or more lines, and 5.00% exhiubited no change compared to the baseline.

### Predictability

A scatter plot with the best-fit line of the attempted versus the achieved SE correction is shown in Figure 2D. Three years postoperatively, the best mimic curve was y = 0.9751x + 0.001. Three years postoperatively, 78.00% of the eyes showed a manifest SE within ±0.50 D of the target; 95.00% were within ±1.00 D of the target (Figure 2E).

### Stability

The manifest SE decreased from −8.94 ± 2.72 D preoperatively to 0.06 ± 0.24 D at 1 month and − 0.36 ± 0.46 D at 3 years postoperatively (Figure 2F). The difference in SE from 1 month to 3 years postoperatively was −0.30 ± 0.37 (−1.13–0.50) D (*p* < 0.05).

### Refractive astigmatism vector analysis

**Figure 3:**
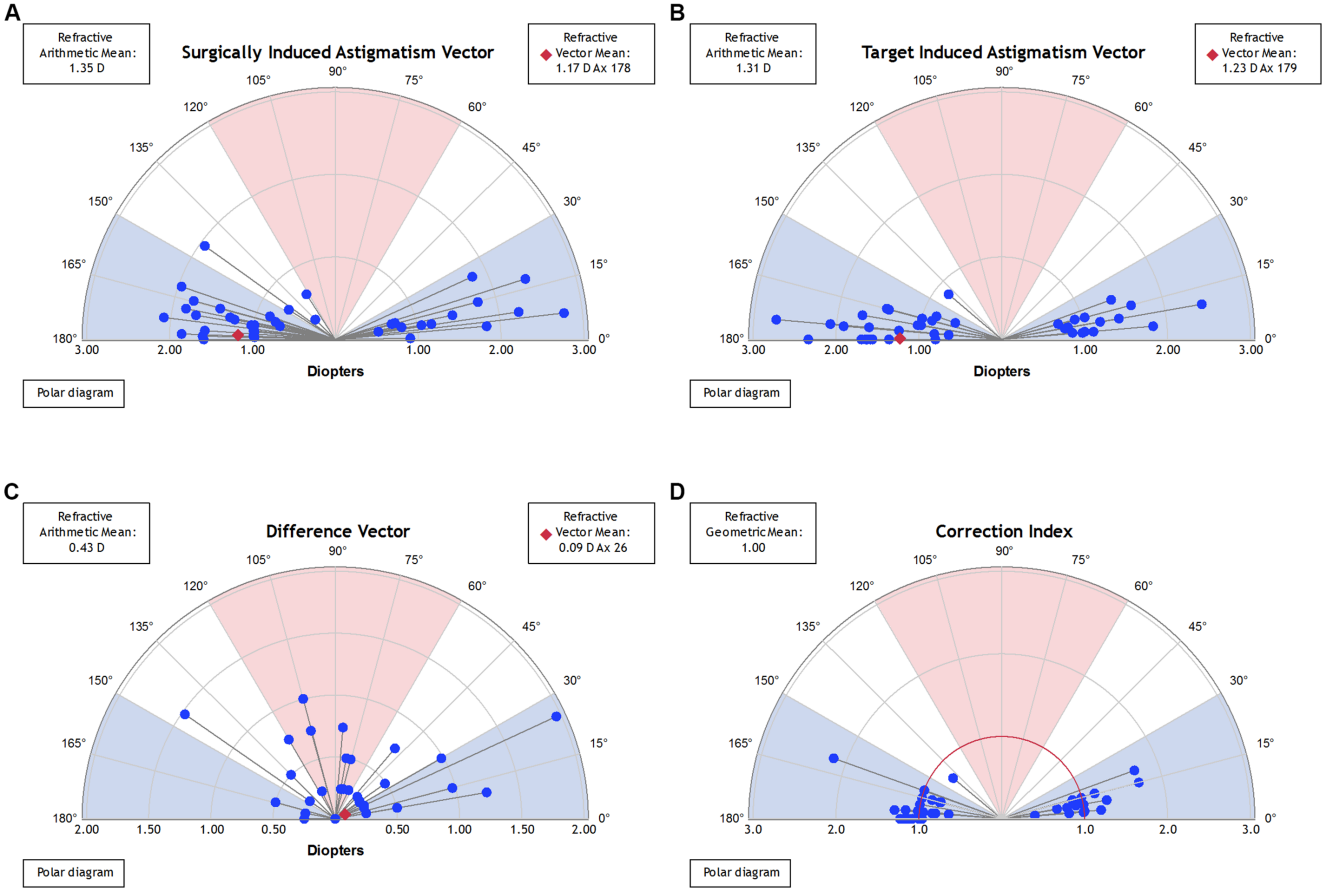
The standard display of polar graphs for surgically induced astigmatism vector **(A)**, target induced astigmatism vector **(B)**, difference vector **(C)**, and correction index **(D)**.

After 3 years, the manifest cylinder was −0.44 ± 0.45 D (range: -2.00–0.00 D) and 75% of eyes were within −0.50 D (Figure 2G). Using Alpin vector analysis, preoperative and postoperative refractive astigmatism were evaluated (10, 11). The mean target induced astigmatism and surgically induced astigmatism were 1.31 ± 0.52 D and 1.35 ± 0.58 D (Figures 2H, 3A,B). The mean arithmetic and absolute angle of error was −3.8 ± 12.0° and 7.2 ± 10.3°(Figures 2I). The mean difference vector and correction index were 0.43 ± 0.44 D and 1.04 ± 0.31 D (Figures 3C,D).

### Rotational stability of TICL

Figure 2; Table 2 show the changes in the angle of the toric ICL and the absolute degree of rotation of the toric ICL from 1 month to 3 years. The mean expected axis of the TICL was −1.15 ± 9.07° (−21–19°) and the mean actual axis of the TICL were − 0.70 ± 9.86 (−20–20°) and − 0.35 ± 11.72 (−25–30°) at 1 month and 3 years postoperatively, respectively. The absolute degrees of all rotations were 3.70 ± 4.42 (0–22°) and 6.00 ± 6.70 (0–32°) 1 month and 3 years postoperatively, respectively (*p* < 0.001). The proportions of absolute rotation angles above 15° at 1 and 3 years postoperatively were 2.5 and 7.5%, respectively. Clockwise rotation occurred in 35.0 and 42.5% of eyes, counterclockwise rotation occurred in 42.5 and 47.5% of eyes, and no rotation occurred in 22.5 and 10% eyes at 1 month and 3 years postoperatively, respectively, compared to the baseline (*p* > 0.05). The mean attempted cylindrical refractive error was −0.10 ± 0.12 D and the mean achieved cylindrical refractive errors were − 0.21 ± 0.30 D and − 0.44 ± 0.45 D at 1 month and 3 years postoperatively, respectively.

**Table 2:** Rotational stability from short to long term in eyes undergoing TICL implantation for myopic astigmatism.

Parameter	1 month	3 years	*p* value
Expected axis of TICL (°)	−1.15 ± 9.07(−21 ~ 19, 40)	−1.15 ± 9.07(−21 ~ 19, 40)	/
Actual axis of TICL (°)	−0.70 ± 9.86(−20 ~ 20, 40)	−0.35 ± 11.72(−25 ~ 30, 40)	0.643
Clockwise rotation (°)	4.64 ± 3.61 (1 ~ 15, 14)	6.12 ± 5.53 (2 ~ 25, 17)	0.398
Counterclockwise rotation (°)	4.88 ± 5.19 (1 ~ 22, 17)	7.16 ± 7.79 (1 ~ 32, 19)	0.316
No rotation (°)	0.00 ± 0.00 (0 ~ 0, 9)	0.00 ± 0.00 (0 ~ 0, 4)	/
Absolute degree of all rotations (°)	3.70 ± 4.42 (0 ~ 22, 40)	6.00 ± 6.70 (0 ~ 32, 40)	0.001
Attempted cylindrical refractive error (D)	−0.10 ± 0.12 (0.00 ~ −0.59, 40)	−0.10 ± 0.12 (0.00 ~ −0.59, 40)	/
Achieved cylindrical refractive error (D)	−0.21 ± 0.30 (0.00 ~ −1.25, 40)	−0.44 ± 0.45 (0.00 ~ −2.00, 40)	<0.001

TICL, Toric implantable collamer lens; D, diopters. Results are expressed as means ± standard deviation (range, number).

Figure 3; Table 3 show the changes in the angle of the toric ICL and the absolute degree of rotation of the toric ICL between low (<2D) and high (≥2D) astigmatic TICL 3 years postoperatively. The mean absolute degrees of rotation that did not cause postoperative astigmatism were 3.73 ± 2.69° and 1.67 ± 1.66° between low (<2D) and high (≥2D) astigmatic TICL (*p* < 0.05), respectively. The ranges were 0–9° and 0–5°, respectively.

**Table 3:** Rotational stability between low and high astigmatic TICL implantation for myopic astigmatism at 3 years.

Parameter	Low astigmatic TICL (<2D)	High astigmatic TICL (≥2D)	*p* value
Clockwise rotation (°)	4.67 ± 2.50 (2 ~ 9, 14)	7.75 ± 7.55 (2 ~ 25, 8)	0.265
Counterclockwise rotation (°)	7.79 ± 8.78 (1 ~ 32, 9)	5.40 ± 4.16 (1 ~ 11, 5)	0.572
No rotation (°)	0.00 ± 0.00 (0 ~ 0, 1)	0.00 ± 0.00 (0 ~ 0, 3)	/
Absolute degree of all rotation (°)	6.29 ± 7.06 (0 ~ 32, 24)	5.56 ± 6.32 (0 ~ 25, 16)	0.741
No postoperative astigmatism (≥ − 0.25D)	3.73 ± 2.69 (0 ~ 9, 15)	1.67 ± 1.66 (0 ~ 5, 9)	0.029
Postoperative astigmatism (<−0.25D)	10.56 ± 9.90 (4 ~ 32, 9)	10.57 ± 6.65 (5 ~ 25, 7)	0.997

TICL, Toric implantable collamer lens; D, diopters. Results are expressed as means ± standard deviation (range, number).

## Discussion

In this prospective study, we analyzed the visual and refractive outcomes after implantation of a EVO-TICL in 40 eyes of 22 patients. We demonstrated that the EVO-TICL is predictable, safe, and effective for correcting low and high levels of astigmatism. Furthermore, EVO-TICL showed promising results in terms of postoperative rotational stability. Although several articles (12–14) have reported the efficacy and safety of toric ICL implantation for myopic astigmatism correction, our study is the first to prospectively investigate the long-term efficacy, safety, and rotational stability and the effect of rotation on astigmatism of the EVO-TICL.

The present study’s safety and efficacy indices were 1.32 and 1.01, respectively, which indicates that TICL implantations offer excellent safety and efficacy in the long term postoperatively. Previous research reported a postoperative safety index of 1.22 and an efficacy index of 1.03 at the 4-year follow-up (15), which is consistent with those reported here. In recent years, multiple research centers have consistently demonstrated ICL’s long-term clinical safety and efficacy in recent years. At the 3-year follow-up visits, 97.50 and 65.00% of the eyes achieved UDVA of 20/40 and 20/20 or better, respectively. Of the eyes, 55.00% gained one line of CDVA, 40.00% of eyes gained two or more lines, and 5.00% of eyes did not change compared to baseline.

The refractive outcomes in this study showed satisfactory predictability. Three years postoperatively, 78.00 and 95.00% of eyes were within ±0.50 D and ± 1.00 D of emmetropia, respectively, and conformed with previous research (16). The manifest SE from 1 month to 3 years postoperatively showed a myopia progression trend, the difference was −0.30 ± 0.37 (−1.13–0.50) D. Yang et al. also reported average SE powers of 0.10 D at 3 months, which progressed to −0.20 D at the 4-year postoperative follow-up (16), which agrees with our results. Lee et al. (17) observed a myopia progression of 0.04 D and 0.02 mm axial elongation per year, and a myopia incidence of 14% from age 20 to 28 years. Similarly, Chen et al. (18) demonstrated that patients with higher myopia and longer AL were more likely to experience continuous axis elongation and progression of myopia. In some cases, the residual SE is up to −1.13 D and patients are unsatisfied with the UDVA, a laser touch-up may be effective and safe after ICL implantation.

The rotational stability of the toric ICL is a crucial factor for achieving high efficacy in the correction of astigmatism. In our study, the absolute degree of rotation at 1 month postoperatively was 3.70 ± 4.42°, which is comparable to that obtained in recent studies evaluating the EVO-TICL (3.75 ± 2.93° and 3.87 ± 3.07° at 3 and 6 months postoperatively, respectively) (8). However, the absolute degree of rotation 3 years postoperatively was 6.00 ± 6.70° in our study. Thus, rotation of the TICL may occur in the early postoperative period or in the long term, and a longer postoperative period correlates with a greater angular variability of TICL rotation.

In our study, we measured postoperative rotation by determining the angle between the adjusted and alignment axes using an image obtained from the OPD-Scan III. Compared with the traditional method of observing the TICL axial position with a slit lamp after mydriasis, measuring the TICL axial position with OPD-Scan III did not require mydriasis and could quickly locate the TICL axial position. The patient did not experience photophobia or discomfort of mydriasis (19).

In this study, clockwise rotation occurred in 35.0 and 42.5% and counterclockwise rotation occurred in 42.5 and 47.5% of eyes at 1 month and 3 years, respectively. Both clockwise and counterclockwise rotations were possible and random. Furthermore, if the TICL rotated 1 month postoperatively, it continued to rotate in the distant 3 years. At 3 years postoperatively, only four eyes did not rotate compared with the preoperative design axes, and 90% of eyes had TICL rotation. In summary, rotations after TICL implantation were within a small range, with individual cases rotating up to 32°. Low astigmatic TICL rotation within 10° did not generate residual astigmatism and UDVA loss. However, high astigmatic TICL rotation outside 5° might cause unwanted astigmatism and UDVA loss. Clearly, rotation in TICL with higher astigmatism had a greater influence on UDVA.

Three years postoperatively, three eyes had a rotation of >15°. Based on the patient’s preoperative white-to-white, sulcus-to-sulcus, and ACD parameters, the slightly smaller ICL size might have caused the significant rotation. The postoperative astigmatism of three eyes was −1.00, −1.50, −2.00 D, respectively, and the patients’ UDVA was 20/30–20/25. The patients experienced UDVA loss, but they thought this had no impact on their daily lives. After fully informed of the pros and cons of the realignment operation, the patients chosed to undergo close monitoring instead of a second operation.

This study had some limitations. First, due to the epidemic’s impact, the 3-year loss to follow-up was high, resulting in an inadequate sample size; we can conduct future studies with a larger sample size over a longer follow-up period. Second, we did not measure the immediate postoperative TICL axis; therefore, perioperative TICL misalignment might have caused the postoperative axis deviation from the expected axis. The surgeon in this study was one of the most experienced surgeons worldwide, so we assumed that all TICLs were placed in the expected axial position.

In conclusion, EVO-TICL implantation is safe and effective, with good predictability and stability. OPD-Scan is a fast device to detect the axial position of the TICL without mydriasis, and the axial position is relatively stable in the long term postoperatively. A slight rotation of low-astigmatic TICL may not cause postoperative astigmatism, whereas rotation of the high-astigmatic TICL may cause it.

## Data availability statement

The raw data supporting the conclusions of this article will be made available by the authors, without undue reservation.

## Ethics statement

This study adhered to the tenets of the Declaration of Helsinki and was approved by the Ethical Committee Review Board of Fudan University Eye and ENT Hospital (2016038). The patients/participants provided their written informed consent to participate in this study.

## Author contributions

XC, HM, MC, I-CL, BL, YJ, YL, XW, and XZ involved in the conception or design of the work, the acquisition, analysis or interpretation of data for the work and agreement to be accountable for all aspects of the work in ensuring that questions related to the accuracy or integrity of any part of the work are appropriately investigated and resolved. XC and HM drafted the work or revising it critically for important intellectual content. XW and XZ final approval of the version to be published. All authors contributed to the article and approved the submitted version.

## Funding

This study was supported by National Natural Science Foundation of China (grant nos. 81770955 and 82171095), Project of Shanghai Science and Technology (grant nos. 20410710100 and 19140900700), Clinical Research Plan of SHDC (grant no. SHDC2020CR1043B), Project of Shanghai Xuhui District Science and Technology (grant no. 2020-015, XHLHGG202104), Shanghai Engineering Research Center of Laser and Autostereoscopic 3D for Vision Care (grant no. 20DZ2255000), Construction of a 3D digital intelligent prevention and control platform for the whole life cycle of highly myopic patients in the Yangtze River Delta (grant no. 21002411600).

## Conflict of interest

The authors declare that the research was conducted in the absence of any commercial or financial relationships that could be construed as a potential conflict of interest.

## Publisher’s note

All claims expressed in this article are solely those of the authors and do not necessarily represent those of their affiliated organizations, or those of the publisher, the editors and the reviewers. Any product that may be evaluated in this article, or claim that may be made by its manufacturer, is not guaranteed or endorsed by the publisher.
